# Appropriateness of rivaroxaban and apixaban dosing in hospitalized patients with a newly diagnosed nonvalvular atrial fibrillation at a single tertiary hospital

**DOI:** 10.1097/MD.0000000000035058

**Published:** 2023-09-08

**Authors:** Mohannad Alshibani

**Affiliations:** a Faculty of Pharmacy, Department of Pharmacy Practice, King Abdulaziz University, Jeddah, Saudi Arabia.

**Keywords:** apixaban, appropriateness, dosing, nonvalvular atrial fibrillation, rivaroxaban, Saudi Arabia

## Abstract

Possible challenges in dosing non-vitamin K antagonist oral anticoagulants in nonvalvular atrial fibrillation (NVAF) and limited evidence in Saudi Arabia make it difficult to assess their appropriateness. This study aimed to assess the appropriateness of prescribing rivaroxaban and apixaban in hospitalized patients with newly diagnosed NVAF. This single-center, descriptive, retrospective study was conducted at a tertiary hospital in Saudi Arabia between December 2018 and December 2019. The included patients were aged 18 years and older with newly diagnosed NVAF who received either rivaroxaban or apixaban during hospitalization. The primary outcome was the dosing appropriateness of rivaroxaban and apixaban in NVAF based on recent food and drug administration prescribing guidelines. Descriptive statistics including frequencies and percentages as well as mean ± standard deviation was used to summarize the data. Pearson Chi-square was used to test for significant difference in proportions of appropriate and inappropriate dosing. Pearson Correlation was used to test for associations between underdosing and overdosing with other patients characteristics. A priori *P* value < .05 was considered significant throughout. A total of 203 patients were included in our analysis. Majority of the patients {125 (61.6%), *P* = .001} received rivaroxaban. Overall, the dosing appropriateness observed in 143 (70.5%) of the patients who received the rivaroxaban and apixaban was significantly higher than the dosing inappropriateness observed in 60 (29.5%) of the patients who received the same drugs, *P* < .001. Apixaban had the highest proportion of patients, 45 (57.7%) with dosing inappropriateness. Overall, underdosing was the most common dosing inappropriateness observed in 53 (26.1%) of the patients. There was a significant negative correlation between the drugs underdosing and creatinine clearance, r = −0.223, *P* = .001. The findings in our present study showed that majority of the patients received appropriate dosing of rivaroxaban and apixaban in hospitalized patients with NVAF. Healthcare providers should update themselves with the recent dosing recommendations for the non-vitamin K-antagonist oral anticoagulants in NVAF to further improve the dosing appropriateness in hospitalized patients with NVAF.

## 1. Introduction

Atrial fibrillation (AF) is rapid and disorganized atrial activation that leads to impaired atrial function.^[1]^ The most common complications of AF include the risk of developing heart failure and stroke, and it increases the risk of mortality if left untreated.^[2]^ AF is a disease whose incidence increases with age, and it might affect more than 4% of people aged 60 years or older.^[3]^ Additionally, the risk of stroke is increased 6-fold by AF and is associated with increased mortality.^[3]^ Unfortunately, the incidence of AF in Saudi Arabia is still not clear, and the available AF registry in Saudi Arabia revealed that patients with AF in Saudi Arabia tend to be younger in age and have a higher rate of diabetes and rheumatic heart disease than those in developed countries.^[4]^

To decrease the risk of embolic events and ischemic stroke, most patients with a history of nonvalvular atrial fibrillation (NVAF) should receive long-term anticoagulants. Current guidelines from the American Heart Association, American College of Cardiology, Heart Rhythm Society, European Society of Cardiology, and American College of Chest Physicians recommend using the CHA_2_DS_2_-VASc score to determine the need for anticoagulant therapy in patients with NVAF.^[5–7]^ CHA_2_DS_2_-VASc score stands for (Congestive heart failure, Hypertension, Age (≥75 years), Diabetes, previous Stroke/transient ischemic attack (TIA), Vascular disease, Age (65–74 years), and Sex **c**ategory [female]). One point was granted for each risk factor except 2 points for age ≥75 years, and the history of stroke/TIA. Current guidelines suggest that anticoagulation should be initiated in patients if the CHA_2_DS_2_-VASc score is at least 2 points for males and 3 points for females.

It is widely known that non-vitamin K antagonist oral anticoagulant (NOACs) dosing in AF might be challenging, and inappropriate NOACs dosing may cause significant drawbacks.^[8,9]^ Each NOACs has some specific criteria, which include renal function, age, weight, and concurrent medications, thus resulting in a more complicated dosing regimen. A recent meta-analysis found that an overdose of NOACs can increase the risk of major bleeding, while an underdose can increase the risk of all-cause mortality with no effect on ischemic stroke.^[10]^

Rivaroxaban, is factor Xa inhibitor, has been approved to decrease the risk of stroke and systemic embolism in NVAF at a dose of 20 mg orally once daily in patients with a creatinine clearance (CrCl) >50 mL/min, and a dose of 15 mg orally once daily in patients with a CrCl ≤ 50 mL/min. The package insert of rivaroxaban recommends avoiding concomitant administration of known combined p-glycoprotein (P-gp) and strong CYP3A4 inhibitors such as ketoconazole, itraconazole, and ritonavir. In addition, concomitant administration of rivaroxaban with known combined P-gp and strong CYP3A4 inducers such as carbamazepine, phenytoin, rifampin, and St. John wort should be avoided.^[11]^

Apixaban, another factor Xa inhibitor, has been approved to reduce the risk of stroke and systemic embolism in NVAF at a dose of 5 mg administered orally twice a day. On the other hand, apixaban dose should be adjusted to 2.5 mg orally twice a day if patients meet at least 2 of the following criteria: 80 years of age or older, body weight 60 kg or less, and serum creatin level 1.5 mg/dL or more. The apixaban package label recommends reducing the dose by 50% for patients receiving an apixaban dose of 5 mg when co-administered with combined P-gp and strong CYP3A4 inhibitors such as ketoconazole, itraconazole, and ritonavir, and avoiding coadministration for patients receiving an apixaban dose of 2.5 mg. Additionally, concomitant administration of apixaban with known combined P-gp and strong CYP3A4 inducers such as carbamazepine, phenytoin, rifampin, and St. John wort should be avoided.^[12]^

Little evidence exists regarding the prescription patterns of NOACs for NVAF in Saudi Arabia. Therefore, this study aimed to assess the appropriateness of prescribing rivaroxaban and apixaban in admitted patients with newly diagnosed NVAF in a single tertiary hospital in Saudi Arabia.

## 2. Methods

In this descriptive retrospective single-center study, data were collected from King Abdulaziz University Hospital from December 2018 to December 2019. The patients who were 18 years and above, hospitalized with a newly confirmed diagnosis of NVAF, and prescribed rivaroxaban or apixaban as anticoagulation therapy for NVAF were included in the study. Patients who were receiving rivaroxaban and apixaban for reasons other than NVAF; dialysis and active bleeding at admission as well as those with missing information were excluded from the study.

The data collected from the patients records included age (years), sex, weight in kg, height (cm), platelet level, hemoglobin level, medication strength, dose, date of the initial dose, frequency, route of administration, concurrent medications, and concurrent diseases. Creatinine clearance was calculated using the Cockcroft–Gault equation^[13]^ when medication was ordered for the first time. Google Sheet and Microsoft Excel version 16.43 were used for data entry. The primary outcome was dosing appropriateness of rivaroxaban and apixaban in NVAF based on recent food and drug administration (FDA) prescribing guidelines.

The appropriateness of dosing and frequency was assessed based on recent FDA prescription guidelines.^[11,12]^ In this study, rivaroxaban dosing was considered appropriate when given at a dose of 20 mg orally once daily in patients with a creatinine clearance (CrCl) > 50 mL/min, and a dose of 15 mg orally once daily in patients with a CrCl ≤ 50 mL/min.^[11]^

Apixaban dosing was considered appropriate when given at a dose of 5 mg orally twice a day and adjusted to 2.5 mg orally twice a day if patients meet at least 2 of the following criteria: 80 years of age or older, body weight 60 kg or less, and serum creatin level 1.5 mg/dL or more.^[12]^

Descriptive statistics including mean and standard deviation for continuous variables, median and interquartile range (IQR) for non-normally distributed variables, and frequencies and percentages for categorical variables were used to summarize the data. Pearson Chi-square was used to test for significant difference in proportions of appropriate and inappropriate dosing. Pearson Correlation was used to test for associations between underdosing and overdosing with platelets counts, creatinine clearance, CHA2 DS2-VACs and hypertension, abnormal renal and liver function, stroke, bleeding, labile INR, elderly, drugs or alcohol (HAS-BLED) Scores. A priori *P* value < .05 was considered significant throughout. Statistical analysis was performed using the Statistical Package for the Social Sciences Version 24, version 24 (IBM, Chicago, IL).

Ethical approval was obtained from the Unit of Biomedical Ethics at King Abdulaziz University before conducting data collection (Reference No 82–21). The requirement for participants consent for this study was waived because no individuals were identified. This study was conducted in accordance with the principles of the Declaration of Helsinki.

## 3. Results

**Figure 1.**
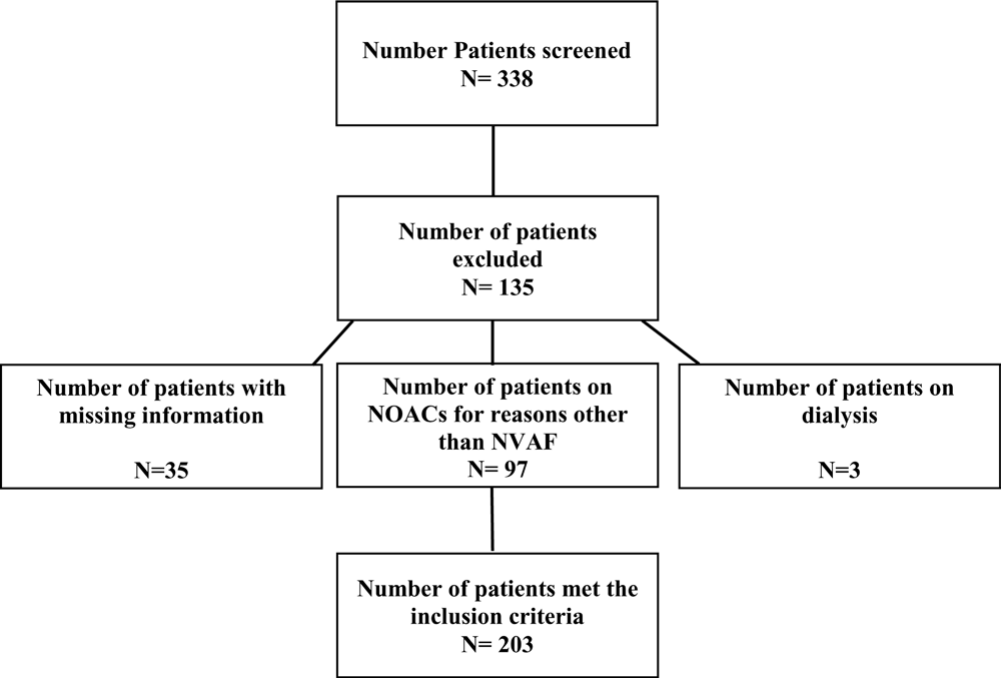
Patients flowchart.

A total of 338 patients were screened during the study period, of whom 203 patients met the inclusion criteria and were included in the final analysis (see Fig. 1). The patient characteristics are presented in Table 1. The mean age of study subjects was 66.3 ± 11.8 years, and the mean weight was 82.9 ± 19.0 kg. The demographic and clinical characteristics of the patients were summarized in Table 1. Majority of the patients were female, 123 (60.6%) and 119 (58.6%) were Saudis. The median serum creatinine was 0.9 ± (0.7–1.2) mg/dL, and the median creatinine clearance was 66.8 ± (48.1–83.2) mL/min. The mean CHA_2_DS_2_-VASc and HAS-BLED scores were 4.18 ± 1.96 and 2.04 ± 1.15, respectively.

**Table 1:** Demographic and clinical characteristics of the patients.

Variable	N = 203	Values
Age, yr (mean ± SD)		66.3 ± 11.8
Weight (kg), (mean ± SD)		82.9 ± 19.0
Height, cm, (mean ± SD)		160.8 ± 8.8
Gender, n (%)		
Male		80 (39.4%)
Female		123 (60.6%)
Nationality, n (%)		
Saudi		119 (58.9%)
Non-Saudi		83 (41.1%)
Serum creatinine, mg/dL, (median ± IQR)		0.9 ± (0.7–1.2)
CrCl, mL/min, (median ± IQR)		66.8 ± (48.1–83.2)
Platelets, ×10^9^/L, (median ± IQR)		266 ± (190.0–319.0)
Hemoglobin, g/dL, (median ± IQR)		12.1 ± (11.2–13.4)
CHA_2_DS_2_-VASc score, (mean ± SD)		4.18 ± (1.96)
HAS-BLED score, (mean ± SD)		2.04 ± (1.15)
Concurrent use of antiplatelets, n (%)		41 (20.3%)
Major Drug-drug interactions, n (%)		2 (0.9%)

CHA_2_DS_2_VACs = congestive heart failure, hypertension, age (≥75 yr), diabetes, previous stroke/transient ischemic attack (TIA), vascular disease, age (65–74 y), and sex category (female), cm = centimeter, CrCl = creatinine clearance, HAS-BLED = hypertension, abnormal renal and liver function, stroke, bleeding, labile INR, elderly, drugs or alcohol, IQR = interquartile range, kg = kilogram, N = number, SD = standard deviation.

Hypertension and diabetes mellitus were the most common comorbid diseases observed in 155 (76.4%) and 128 (63.1%) patients, respectively. Details of the other comorbid diseases can be seen in Table 2.

**Table 2:** Patients concurrent diseases.

Concurrent disease	N = 203	n (%)
Heart failure		95 (46.8%)
Hypertension		155 (76.4%)
Diabetes mellitus		128 (63.1%)
Stroke		46 (22.8%)
Peripheral artery disease		47 (23.2%)
Chronic kidney disease		5 (2.5%)

Majority of the patients {125 (61.6%), *P* = .001} received rivaroxaban. Overall, the dosing appropriateness observed in 143 (70.5%) of the patients who received the rivaroxaban and apixaban was significantly higher than the dosing inappropriateness observed in 60 (29.5%) of the patients who received the same drugs, *P* < .001. Apixaban had the highest proportion of patients, 45 (57.7%) with dosing inappropriateness. Overall, underdosing was the most common dosing inappropriateness observed in 53 (26.1%) of the patients (Table 3).

There was a significant negative correlation between the drugs underdosing and creatinine clearance, r = −0.223, *P* = .001. On the other hand, the underdosing correlates positively and significantly with the age of the patients, *R* = 0.250, *P* < .001. Other details of the associations between underdosing and overdosing with platelets counts, creatinine clearance, CHA2DS_2_-VACs Score, HAS-BLED Score, age and body weight can be seen in Table 4.

**Table 3:** Dosing appropriateness.

Overall dosing	N = 203	n (%)	*P* value
Appropriate dose		143 (70.5%)	<.001
Underdose		53 (26.1%)	
Overdose		7 (3.4%)	
Dosing in rivaroxaban, n = 125			
Appropriate dose		110 (88.0%)	.001
Underdose		10 (8.0%)	
Overdose		5 (4.0%)	
Dosing in apixaban, n = 78			
Appropriate dose		33 (42.3%)	
Underdose		43 (55.1%)	
Overdose		2 (2.6%)	

**Table 4:** Correlation: associations between underdosing and overdosing with platelets counts, creatinine clearance, CHA_2_DS_2_-VACs Score, HAS-BLED Score, age and body weight.

Variables		Underdosing	Overdosing	Platelets counts	Creatinine clearance	CHA_2_DS_2_-VACs Score	HAS-BLED Score	Age of the patients	Body weight (kg)
Underdosing	Corr. Coef.	1.000							
	*P* value								
Overdosing	Corr. Coef.	−0.112	1.000						
	*P* value	0.111							
Platelets counts	Corr. Coef.	−0.027	0.105	1.000					
	*P* value	0.730	0.175						
Creatinine clearance	Corr. Coef.	−0.223†	−0.155*	0.029	1.000				
	*P* value	0.001	0.028	0.707					
CHA_2_DS_2_VACs Score	Corr. Coef.	0.209†	0.124	−0.103	−0.356†	1.000			
	*P* value	0.003	0.085	0.190	0.000				
HAS-BLED Score	Corr. Coef.	0.281†	0.115	−0.126	−0.455†	0.756†	1.000		
	*P* value	0.000	0.109	0.107	0.000	0.000			
Age of the patients	Corr. Coef.	0.250†	0.131	−0.155*	−0.504†	0.600†	0.564†	1.000	
	*P value*	0.000	0.062	0.044	0.000	0.000	0.000		
Body weight (kg)	Corr. Coef.	−0.081	0.063	−0.099	0.362†	0.073	0.043	−0.134	1.000
	*P* value	0.249	0.372	0.198	0.000	0.313	0.553	0.056	

CHA_2_DS_2_VACs = congestive heart failure, hypertension, age (≥75 yr), diabetes, previous stroke/transient ischemic attack (TIA), vascular disease, age (65–74 yr), and sex category (female), HAS-BLED = hypertension, abnormal renal and liver function, stroke, bleeding, labile INR, elderly, drugs or alcohol.

* Correlation is significant at the 0.05 level (2-tailed).

† Correlation is significant at the 0.01 level (2-tailed).

## 4. Discussion

This single-center, retrospective descriptive study evaluated the appropriateness of rivaroxaban and apixaban dosing in hospitalized patients with newly diagnosed NVAF in Saudi Arabia. The main finding of this study is the high incidence of inappropriate NOACs dosing, which confirms the complexity associated with NOACs regimens. To the best of our knowledge, this is the first study to examine the appropriateness of rivaroxaban and apixaban dosing for NVAF in Saudi Arabia. The inappropriateness of rivaroxaban and apixaban prescriptions in this study was 29.6%, which was similar to another single-center retrospective cohort study conducted in the Eastern Region of Saudi Arabia, which found that the reduced dose of dabigatran 110 mg for patients with NVAF was inappropriately prescribed to approximately 31% of the included subjects.^[14]^

However, the inappropriateness of this study was higher than that of another single-center retrospective observational cohort study in Ireland, in which they identified that the inappropriateness of rivaroxaban, dabigatran, and apixaban dosing in NVAF patients was 20.4%. This difference between the 2 studies may be due to better experience of recent prescribing guidance. It should be noted that our study followed the FDA prescribing guidance for NOACs dosing, while another study followed the recommendations of the European Medicines Agency to evaluate the appropriateness of NOACs.^[15]^

In this study, there was a higher inappropriate dose of apixaban (57.7%) than rivaroxaban (12%). This finding was similar to that of another single-center retrospective cohort study in Canada, in which they included 47 patients and found that the dosing of apixaban was inappropriate in 53% of patients with NVAF. It should be noted that later studies have evaluated the appropriateness of apixaban dosing in relation to the ARISTOTLE study.^[16]^ Another single-center retrospective cohort study in the Netherlands included 3231 patients with NVAF and identified that among the different NOACS that were prescribed, apixaban was prescribed to 28.3%, and it was most frequently inappropriate in 41.4% of the included patients.^[9]^

It should be noted that most of the inappropriate dosing was related to underdosing either in apixaban (55.1%) or rivaroxaban (8%). The issue of underdosing of NOACs is global, and it might be explained by the fact that prescribers believe this might decrease the risk of bleeding without paying attention to the risk of ischemic stroke and all-cause mortality due to underdosing.^[10,17,18]^ A single-center retrospective cohort study conducted in a teaching hospital in the Netherlands to evaluate the appropriateness of NOACs dosing in 3231 hospitalized patients with NVAF found that patients who were prescribed dabigatran, rivaroxaban, or apixaban received the reduced dose inappropriately in 5.4% compared to 4.5% who received overdose.^[9]^ Additionally, another single-center retrospective cohort study in Japan included 316 patients to investigate NOACs appropriateness in NVAF patients revealed that patients who were prescribed dabigatran, rivaroxaban, apixaban, or edoxaban received the reduced dose inappropriately in 19.3% compared to only 2.5% received overdose.^[19]^

The findings of this study are quite interesting because little is known about the prescribing patterns of NOACs in NVAF in Saudi Arabia, and the results of this study could bring more attention to prescribing and monitoring within any healthcare system. The high number of inappropriate NOACs prescriptions highlights the urgent need for pharmacovigilance and follow-up after discharge. In addition, the role of a multidisciplinary team, including physicians and clinical pharmacists, is crucial for detecting dosing errors or drug-drug interactions during hospitalization. Preferably, patients medical history, concurrent medications, and recent laboratory data should be available in shared electronic systems and accessible to different healthcare systems in order to decrease drug duplication and minimize dosing error.

This study had several limitations that should be considered. First, it should be noted that the retrospective nature of this study might limit all eligible patients with NVAF and decrease the generalizability of our findings. Second, it was not the focus of this study to evaluate any clinical outcomes, which might be important in such aspects. Third, the availability of only 2 NOACs at our institution might limit the options for prescribers to choose among the available NOACs in practice.

## 5. Conclusion

The findings in our present study showed that majority of the patients received appropriate dosing of rivaroxaban and apixaban in hospitalized patients with NVAF. Healthcare providers should update themselves with the recent dosing recommendations for the NOACs in NVAF to further improve the dosing appropriateness in hospitalized patients with NVAF.

## Acknowledgments

I would like to thank Alyaa Alrifai, Wed Sairafi, and Lujain Masoudi for their help with the data collection.

## Author contributions

**Conceptualization:** Mohannad Alshibani.

**Data curation:** Mohannad Alshibani.

**Formal analysis:** Mohannad Alshibani.

**Methodology:** Mohannad Alshibani.

**Writing – original draft:** Mohannad Alshibani.

**Writing – review & editing:** Mohannad Alshibani.
